# 

**Published:** 1890-07-19

**Authors:** 


### NOTICE TO CORRESPONDENTS.

All MS., Letters, Books for Review, and other matters intended for the Editor should be addressed The Editor, The Lodge, Porchester Square, London, W.

The Editor cannot undertake to return rejected M.S., even when accompanied by stamped directed envelope.

### Notice to Advertisers and Subscribers.

All Advertisements, Orders for Copies of The Hospital, and Business Communications should be addressed to The Publisher, *not to the Editor*, The Hospital, 140, Strand, W.C.

### Bound Volumes of "The Hospital."

Vol. VI., containing full page portraits of T.R.H. the Prince and Princess of Wales, and Miss Florence Nightingale, is obtainable at 4s., post free.

Orders will be received for Vol. VII., 4s. post free. Cases for binding may be had of the Publisher, at 1s. 6d. each.

### To Residents, Secretaries, and Matrons.

The Editor will be glad to have the Regulations and each Report as issued by Asylums, Hospitals, Convalescent and Nursing Institutions, as well as those of other Charities, and an early copy in duplicate of all Appeals and Festival Notices, etc., addressed to *The Editor, The Lodge, Porchester Square, London, W.* Any superintendent, secretary, matron, or other chief official who regularly sends such information may be registered, on application to the Publisher, to receive free of cost a cover for binding The Hospital in half-yearly volumes.

### BURDETT'S HOSPITAL ANNUAL FOR 1890

Is now ready, and may be obtained of the Publisher, The Hospital Offices, 140, Strand, W.C., price 2s. 6d. limp boards, or 3s. 6d. cloth. All orders must be accompanied by a remittance.

### Scraps and Gleanings.

London working boys are thoroughly enjoying the Seaside Camp pitched again this year at Deal.

The leprosy plague in Noumea has abated since steps have been taken to cut off the supply of alcohol to the aborigines.

A meeting was held at the Mansion House, on July 3rd, in support of the International Congress of Hygiene and Demography, 1891.

A very pleasant *Soirée Musicale* in aid of St. John's Hospital for Diseases of the Skin, took place at the Prince's Hall last week.

C. G. Robson Scott, M.B. Edin., M.R.C.S., L.R.C.P. Lond., has been appointed house physician for six months, commencing July 1st, 1890.

Mr. West, of London, has been elected as Foreign Associate of the French Academy of Medicine. He had been a correspondent since 1875.

Clayton has got a new secretary for its Hospital Saturday Fund; let us hope he will do as well as the old one. Over £1,000 was collected last year.

Dr. Wilks, of Guy's Hospital, and other medical men denounce the detestable decoctions under the name of port frequently used among the sick poor.

Over £200 was taken by collecting boxes for the hospital at Richmond from January to June. We congratulate the hospital on its energetic secretary.

The United Friendly, Temperance, and Trade Societies lately held a grand demonstration in aid of the Children's Hospital, Great Ormond Street. The collection amounted to over £100.

The editor of the *St. James's Gazette* has collected over £2,600 for the relief of the survivors of the Light Brigade charge at Balaclava, many of whom had have to take refuge in the workhouse.

It has been officially notified to the governing body of the University College of North Wales, at Bangor, that the bequest under the will of the late Dr. Evan Williams, of Manchester, amounts to £53,000.

The Marquis of Lothian has visited the Shetland Combination Poorhouse, and expressed his pleasure with the arrangements there. It is a far cry to Shetland! but our sympathy is with our fellow-workers there.

The Rev. Dr. Herman Adler, the acting Chief Rabbi, has remitted to the Lord Mayor the sum of £683 4s. 1d., being the aggregate sum collected in the metropolitan synagogues under his pastoral care for the Hospital Sunday Fund.

The College of Physicians of Edinburgh has awarded to Mr. Lawson Tait a share in the Cullen Jubilee Prize, given for "the greatest benefit done to practical medicine by applying surgical means for the relief of medical cases."

Our sympathy was with the Middlesex Hospital Medical Society on July 4th. What ought to have been one of the most charming receptions, was more or less spoiled by the unfavourable weather. Still, a very pleasant evening was spent.

The bazaar at the Royal Hospital for Incurables was very successful, the presence of the Duchess of Teck attracting a large number of visitors. The money taken at the stall for outsiders was enough to give each patient too ill to work, a small gift.

In the month of June 11,042 tons of fish were delivered at Billingsgate Market, and of this quantity 54 tons 11 cwt. were seized by the officers of the Fishmongers' Company as unfit for food. The condemned fish included 16 tons of plaice, all immature fish.

Mr. R. T. Williamson, M.D. (Lond.), M.R.C.P. (Lond.), B.S., M.R.C.S., L.S.A., has been appointed honorary medical officer to the Pendleton Branch Dispensary of the Salford Royal Hospital, in succession to A. Jasper Anderson, M.A., M.B. (Oxon), resigned.

Mrs. St. A. Horton, the hon. secretary of the St. Raphael's Hospital for Consumptives, Worthing, wishes it known that the London, Brighton, and South Coast Railway now conveys patients between London and Worthing (and *vice versa*) at the reduced fare of 5s. the double journey. The passes were granted from July 1st.

Deputy Inspector General of Hospitals and Fleets (W. H. Adam, R.N.), has accepted the office of assistant association secretary of the Missions to Seamen for the Midland counties, for the provision of chaplains and readers for the shipping, fishing vessels, ande barges, at home and abroad, and for their crews when ashore. His headquarters will be at Birmingham.

